# Adolescents With Labial Enlargement Misdiagnosed as Labial Masses on Imaging

**DOI:** 10.1155/crpe/6823679

**Published:** 2025-05-22

**Authors:** Erin Isaacson, David A. Bloom, Melina L. Dendrinos

**Affiliations:** ^1^Department of Obstetrics and Gynecology, University of Michigan, Ann Arbor, Michigan, USA; ^2^Department of Radiology, University of Michigan, Ann Arbor, Michigan, USA

**Keywords:** aphthous ulceration, asymmetric labia, labia minora enlargement

## Abstract

**Background:** Literature describing radiologic imaging of the adolescent labia is lacking and may lead to misdiagnoses and unnecessary medical care.

**Cases:** Two adolescent patients presented with sudden-onset unilateral labia minora enlargement and underwent multiple imaging modalities which identified a discrete mass. One patient was diagnosed with aphthous ulcers after a prolonged emergency department stay, and the other underwent an exam under anesthesia for mass removal and was found to have an elongated labia minora rolled into itself.

**Conclusion:** Labia minora enlargement in adolescents can be significant and may be mistaken for a discrete mass on radiologic imaging given a lack of literature on expected findings. Clinical correlation and understanding of age-appropriate etiologies of noninfectious labial swelling is crucial to avoid unnecessary medical therapies and procedures.

## 1. Introduction

The current literature related to imaging of the labia and perineum primarily focuses on malignancy and other pathologies that are most prevalent in the adult population, such as benign neoplasms or Bartholin gland cysts and abscesses [1, 2]. Magnetic resonance imaging (MRI) is established as the ideal imaging modality for the perineum given its excellent contrast resolution, which is particularly important when evaluating for the possible extent of a malignancy, but ultrasonography is often obtained first due to cost and availability [3]. However, pathologies in adolescents differ significantly from adults, and practitioners may not be as experienced with labial anatomy or the presentations of the rare conditions seen in this patient population [4, 5]. For example, nontraumatic acute labial enlargement, though commonly caused by infection, may also result from underlying noninfectious inflammatory processes [5]. Additionally, the expected labial and perineal imaging findings in this population, outside of rare masses and vascular malformations, are not well-described in the literature [6–8]. These factors combined may cause patients to be misdiagnosed, leading to unnecessary clinical care or treatments [2].

We present two cases of adolescent patients with acute unilateral labia minora enlargement, where the imaging evaluation diagnosed a labial mass and resulted in unnecessary medical care.

Patients and parents provided written consent for use of medical records and diagnostic imaging.

## 2. Cases

**Figure 1:**
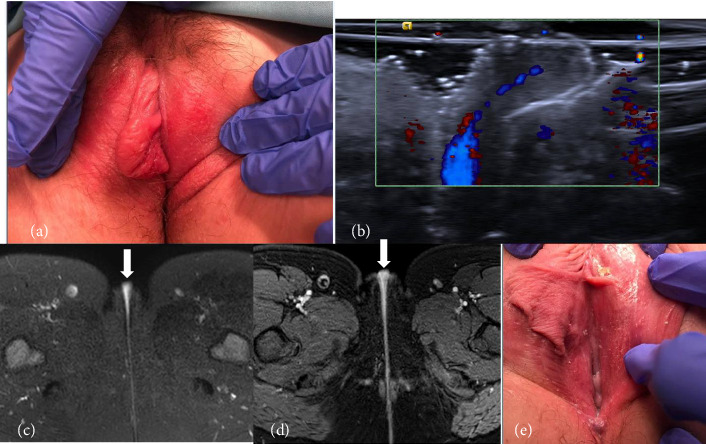
Patient 1 initial examination, imaging (ultrasound and MRI), and intraoperative findings. (a) Outpatient examination revealing an enlarged right labium minus “mass.” (b) Transverse high-resolution ultrasound with color Doppler confirms a hypoechoic solid lesion. (c) Axial T2-weighted MRI demonstrates a hyperintense lesion (arrow). (d) Postcontrast axial T1-weighted images with fat saturation show enhancing labial mass (arrows). (e) Intraoperative findings of an elongated right labium minus.

SV was a 10-year-old postmenarchal female who presented to pediatric and adolescent gynecology clinic with 48 h of perineal irritation and enlargement. A family member in the medical field had noted a 1 × 3 cm “cystic appearing mass” on the vulva. She denied any trauma, recent bicycling or horseback riding, discharge, placement of items into the vagina, or sexual assault. On examination, she was afebrile and nontachycardic. The right labium minus was enlarged with a 1 × 3 cm nonfluctuant, nontender, nonerythematous, somewhat firm mass not continuous with the clitoris, vagina, or perineum (Figure 1(a)). Bilateral labia majora were normal.

Imaging was recommended to further evaluate the labia. Labial ultrasound was performed and reported a soft tissue mass with a vascular pedicle that appeared hyperemic to the adjacent labial tissue with no drainable fluid collection (Figure 1(b)). She then underwent a contrast-enhanced pelvic MRI that reported a 1.9 × 0.9 cm superficial mass appearing to arise from the right vulva, with increased T2 signal and enhancement near midline, concerning for a deeper origin within the vagina (Figures 1(c) and 1(d)). Differential diagnosis included fibroma, neurofibroma, hamartoma, leiomyoma, rhabdomyoma, or hemangioma. Due to continued symptoms and concern for a mass, the patient and family desired surgical removal. She went to the operating room 9 days after her initial visit. External genitalia were normal apart from asymmetric labia minora, with the right labium minus measuring 6 cm wide from the hymenal opening (Figure 1(e)), consistent with a diagnosis of labial hypertrophy. She was managed conservatively and symptoms resolved.

## 3. Case 2

HS was a 13-year-old postmenarchal female who presented to the emergency department (ED) after one day of progressive labial swelling and pain. She had initially noted symptoms while washing the perineal region and was seen by her pediatrician who recommended ice packs. The swelling worsened until it was too painful to walk, which led to her ED visit. She denied sexual activity, trauma, strenuous activity, shaving, or hygiene changes but did report cold-like symptoms for a few days prior to this event. She was afebrile and nontachycardic on presentation.

**Figure 2:**
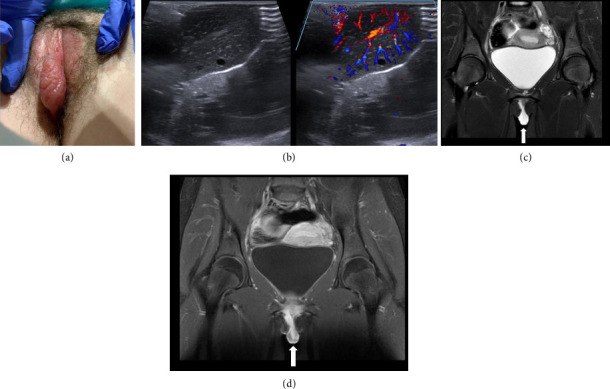
Patient 2 examination and imaging (ultrasound and MRI) findings. (a) Imaging findings on ED presentation. (b) Sagittal high-resolution ultrasound demonstrating a solid right labium mass with hyperemia on color Doppler imaging. (c) Coronal T2-weighted MRI demonstrating a hyperintense right labium mass (arrow). (d) Postcontrast coronal T1-weighted image with fat saturation shows intense heterogeneous enhancement of the mass (arrow).

Initial examination by the gynecology consultant noted diffuse swelling of the right labium minus with exquisite tenderness and mild erythema (Figure 2(a)). On the inferior-medial aspect, a region of dark discoloration was noted but exam was limited due to patient discomfort.

Imaging was requested by the emergency physician, given the significant labial swelling. A targeted ultrasound of the vulva demonstrated a 3.4 × 2.2 × 2.1 cm hypervascular solid mass (Figure 2(b)). An MRI was then obtained, which demonstrated a 4.1 × 1.6 × 2.9 cm pedunculated enhancing mass involving the right labium minus. While an infectious/inflammatory etiology was considered in the imaging report, no significant surrounding inflammatory change was noted in the adjacent subcutaneous tissues. Fibroma, neurofibroma, hemangioma, myofibroblastic tumor, and sarcoma were all considered (Figures 2(c) and 2(d)).

The pediatric gynecology service was then consulted given the unclear diagnosis. On re-examination, a 5 mm ulceration was noted on the right inner labium minus, surrounded by black and blue necrotic-appearing tissue. Given the clinical course, examination findings, and recent viral illness, the patient was diagnosed with vulvar aphthous ulcers. She was discharged after 16 h in the ED and prescribed a 7-day oral steroid taper. She was seen in pediatric gynecology clinic 3 weeks later with complete resolution of symptoms.

## 4. Discussion

The utility of imaging in the work-up of acute labia minora enlargement in adolescents is not well-established, with limited literature on the expected findings. Many providers are unfamiliar with adolescent labial anatomy or the conditions that commonly present in this population, leading to potential misinterpretation and misdiagnosis [2]. In this case series, we present two patients with unilateral labia minora enlargement misdiagnosed with masses after diagnostic imaging that ultimately underwent unnecessary medical care.

Although masses or vascular lesions are in the differential diagnosis when initially considering the etiology of unilateral swelling in the perineal region, they are rare in this age group and are described infrequently in the literature—usually in case reports [4, 9–11]. Additionally, a mass would be very unlikely to present with sudden onset of symptoms as in our patients; often, the presentation is over months or even years [9–11]. Therefore, the importance of a comprehensive clinical evaluation cannot be understated. An initial examination in a case of labial inflammation should include a thorough history of symptom onset and risk factors and exposure to any infectious causes. An external genitalia exam should be performed as tolerated without anesthesia, looking for signs of infection, ulcerations, masses, or excess labial tissue. There are not clear guidelines for pursuing imaging in cases of labial inflammation within the pediatric population given the rarity of masses and therefore should be individualized to each patient.

Patient 1 was ultimately diagnosed with asymmetric labia minora hypertrophy, which is a common complaint in adolescents. “Normal” labial anatomy varies widely in shape and size, and hypertrophy is often subjective; however, it is traditionally diagnosed when the labial length is > 4 cm [12, 13]. Elongated labial tissue can cause pain and irritation, and patients may tuck in the excess tissue to avoid it getting caught in clothing [5, 12, 14]. In our patient, the labial tissue had folded into the vaginal opening and become mildly inflamed, giving the appearance of a possible mass on physical examination. The vascular pedicle depicted on imaging was likely just a branch of the normal blood supply to the labia minora; however, although the supply is known to come from both the external and internal pudendal arteries, the specific vascular anatomy of the labia until recently has been poorly defined, which leads to lack of familiarity and confusion about what constitutes a normal finding [15]. Recent studies have identified that the external and internal pudendal arteries create a fine anastomosing network of vessels leading to the labia minora arteries, but these findings have yet to be widely disseminated [16].

Patient 2 presented with an uncommon condition: labia minora aphthous ulcers. First described by Lipschutz in 1913, this is an acute finding in young women, often associated in conjunction with symptoms of a systemic or flu-like illness [17–19]. The diagnosis can be challenging, as it is one of exclusion and there may only be a single ulceration [18, 19]. Additionally, until recently, most reviews on labial conditions mentioned aphthous ulcers briefly, if at all, leading to a lack of familiarity by many providers [20]. The ulcer is often found on the medial aspect of the labia minora and has been known to present with significant edema of the surrounding soft tissues, as was the case with our patient, or even frank cellulitis. However, there is a lack of published data on the specific criteria for this diagnosis in the clinical setting and on whether there is any utility for imaging in the setting of significant edema [21]. There is no literature on the expected imaging findings in noninfectious labial edema, as imaging is not consistently performed during work-up. In fact, there is only one case report that specifically describes the MRI findings of an adolescent with sudden onset labial edema secondary to rhabdomyolysis [7]. The authors reported finding T2-weighted hyperintensity within the vulvar region, similar to our patient's imaging, and concluded the labial enlargement was edema secondary to the underlying inflammatory process [7]. Only a few case reports are available that correlate the clinical picture of adolescent labial inflammation and/or edema to findings seen on imaging examinations, representing a significant gap in the literature [22].

Pediatric and adolescent vulvar conditions, when exhibiting unique presentations like these two cases, can pose diagnostic challenges. The use of imaging in the work-up of more acute labial enlargement in this population is rarely mentioned in the literature, leading to a broad differential diagnosis and potential for misdiagnosis like those seen in this report. Continued education regarding normal labial anatomy in adolescents and differential diagnoses for labial enlargement, as well as further research examining the role of imaging and expected findings in unique presentations, will mitigate misdiagnosis and unnecessary procedures.

## Data Availability

The data that support the findings of this study are available on request from the corresponding author. The data are not publicly available due to privacy or ethical restrictions.

## Nomenclature

MRI: Magnetic resonance imaging
ED: Emergency department
